# Population density does not influence male gonadal investment in the Least Killifish, *Heterandria formosa*

**DOI:** 10.1002/ece3.402

**Published:** 2012-10-22

**Authors:** Matthew Schrader, Joseph J Apodaca, Pamela S D Macrae, Joseph Travis

**Affiliations:** 1 Department of Biological Science, Florida State UniversityTallahassee, Florida; 2 Department of Animal Biology, University of Illinois Urbana-ChampaignChampaign, Illinois

**Keywords:** *Heterandria formosa*, population density, sperm competition

## Abstract

Comparative studies documenting a relationship between male gonadal investment and the degree of sperm competition (SC) have usually considered the association between these traits to be driven by qualitative differences in the mating system, such as whether spawning occurs in pairs or groups. However, ecological and demographic differences between conspecific populations may also generate variation in the importance of SC that can drive the evolution of male gonadal investment. In this study, we examined whether variation in population density, which is predicted to influence the level of SC in many animals, is correlated with male gonadal investment among populations of the least killifish, *Heterandria formosa,* a species with internal fertilization in which multiple mating is common. We complemented this field study by testing whether males respond plastically to experimentally increased levels of SC by increasing investment in testis. This experiment involved two treatments. In the first, we eliminated the potential for sperm competition (NSC) by housing a single male with a single female. In the second, we created a high risk of SC by housing five males with two females. In the field survey, we found significant differences among populations in density and relative testis mass. However, there was no evidence for a correlation between population density and relative testis mass. In our lab experiment, males did not adjust their gonadal investment in response to experiencing different levels of SC for 4 weeks. Our combined results indicate that gonadal investment in male *H. formosa* is not related to variation in population density.

## Introduction

Sperm competition (SC) occurs when the sperm of two or more males compete over the fertilization of a given set of ova (Parker 1970) and the risk and intensity of SC play important roles in the evolution of mating behaviors, characteristics of gametes and ejaculates, and the morphology of reproductive structures (Birkhead and Moller 1989). One of the major predictions of SC theory is that males should increase their relative investment into ejaculates as the level of SC increases (Parker 1988). This prediction has been tested using three general approaches. First, comparative studies have examined whether interspecific variation in male investment into ejaculates is correlated with the level of SC (Harcourt et al. 1981; Gage 1994; Hosken 1997, 1998; Stockley et al. 1997). In these studies, variation in the level of SC is usually determined by qualitative characteristics of the mating system, such as whether fertilization is internal or external or whether spawning occurs in pairs or groups (Stockley et al. 1997). Second, comparative studies at the intraspecific level have tested whether males facing different risks of SC (e.g., because they adopt different mating strategies) differ in gonadal investment (Taborsky 1994; Petersen and Warner 1998). Finally, experimental evolution has been used to test directly how testis mass evolves when SC is eliminated thorough enforced monogamy (Hosken and Ward 2001; Pitnick et al. 2001; Wigby and Chapman 2004; Crudgington et al. 2009).

The different approaches to testing the prediction have produced uneven results. On the one hand, there is strong support from inter- and intraspecific comparative studies (Harcourt et al. 1981; Hosken 1997, 1998; Stockley et al. 1997). In fact, a positive association between the level of SC and investment in testes is so well accepted that studies often use relative testis mass itself as an index for the level of SC (Long 2005). On the other hand, the results from studies of experimental evolution are mixed. Some studies have found that the removal of SC results in a decline in relative testis mass (Hosken and Ward 2001; Pitnick et al. 2001); other studies have found no such response (Wigby and Chapman 2004; Crudgington et al. 2009).

While comparative studies have exploited qualitative differences in mating systems as sources of variation in the level of SC, ecological and demographic differences between conspecific populations can also create differences in the importance of SC (Gage 1995; Richardson and Burke 2001; Dziminski et al. 2010). For example, several authors have argued that the risk of SC in the Trinidadian guppy, *Poecilia reticulata* varies among populations with different predation regimes that impose different daily mortality rates (Evans and Magurran 1999a; Elgee et al. 2010).

Population density is an ecological variable that often varies considerably among populations and that is likely to influence the level of SC in species ranging from insects to mammals to marine invertebrates (Gage 1995; Long and Montgomerie 2006; Levitan 2008). In territorial species, such as many reef fish, the risk of SC increases with population density because at high densities, territorial males are less effective at defending mating territories and females from nonterritorial males (Petersen and Warner 1998). In nonterritorial species, SC can increase with population density simply because high densities increase encounter rates between males and females, or in the case of externally fertilizing species, their gametes (Levitan 2008). The results of several recent studies are consistent with the hypothesis that population density influences the level of SC. For example, positive correlations between population density and male gonadal investment have been reported in insects, birds, mammals, and amphibians (Gage 1995; Brown and Brown 2003; Long and Montgomerie 2006; Dziminski et al. 2010).

In this study, we combine data from a field study and laboratory experiment to test whether variation in population density influences male gonadal investment in the poeciliid fish, *Heterandria formosa*. We first use data from several *H. formosa* populations to examine whether relative testis mass is correlated with population density. Previous work in this species has shown wide variation among populations in density, and this variation appears to drive the evolution of female life history traits, such as the size and number of offspring (Leips and Travis 1999; Soucy and Travis 2003; Richardson et al. 2006; Schrader et al. 2011; Schrader and Travis 2012). Whether male traits covary with population density has not been assessed. In the second part of this study, we manipulated the level of SC under laboratory conditions to test whether male investment in testes varies plastically with the potential for SC.

## Materials and Methods

**Figure 1:**
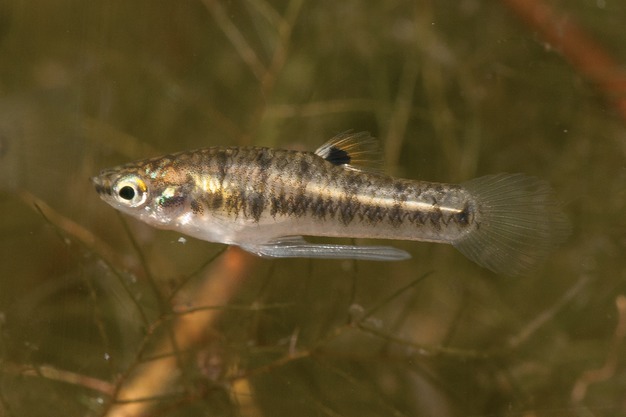
A *Heterandria formosa* male. Photo courtesy of Pierson Hill.

*Heterandria formosa* is a poeciliid fish that occupies a variety of freshwater habitats throughout the coastal plain of the southeastern United States (Baer 1998). Males (Fig. 1) do not display to females, relying instead on gonopodial thrusting during mating (Farr 1989). Gonopodial thrusting is usually referred to as a coercive insemination attempt in which a male orients himself behind a female, swings his intromittent organ (a modified anal fin called the gonopodium) forward, and attempts to insert the gonopodial tip onto the female's gonopore (Farr 1989). The absence of courtship in this species suggests that the level of SC will increase with population density because increases in density produce increases in male-female encounter rates. In addition, females can store sperm for a considerable portion of their lifespan (S. Soucy and J. Travis, unpubl. data) and, at higher densities; females should be accumulating a greater diversity of stored sperm.

We estimated population densities and collected *H. formosa* males from 15 North Florida populations in May 2012. We estimated adult density (adults per 0.5 m^2^) in each population using methods described elsewhere (Leips and Travis 1999; Richardson et al. 2006). Briefly, we employed a stratified sampling procedure in which, at each site, we threw the trap, haphazardly, three times in shallow, nearshore vegetated areas. The contents of each trap throw were removed with 10 sweeps of a dipnet and placed in a water-filled bucket. We then sorted through the contents of each bucket and counted the number of female, male, and juvenile *H. formosa* caught in each trap throw. The adult density at each site was estimated as the average number of adults caught across the three trap throws. Prior work in a subset of these populations has shown that variation in density is consistent over long periods and that these measures of density indicate long-term density regimes, as evidenced by the fact that high density populations harbor more genetic variation (more alleles and higher levels of heterozygosity at microsatellite loci) than low density populations (Soucy and Travis 2003; Schrader et al. 2011).

We attempted to collect 20 males from each population. Males were either caught in the throw-trap while estimating population density or with dipnets. Males were euthanized in the field with an overdose of anesthetic (MS222) and preserved in 10% formalin until they were measured and dissected. In most populations, we were unable to collect 20 males because of low densities. In addition, some of the males we collected were classified as immature after examination of the gonopodium under a dissecting scope. Excluding these immature males further reduced out sample size. In the end, we had between 7 and 18 mature males per population (Table 1).

**Table 1:** Adult densities (May 2012 estimates and long-term estimates), mean body mass (mg), and mean testis mass (mg), in each population sampled in May 2012

	Adult density (adults per 0.5 m^2^)	Mean body mass (SE)	Mean testis mass (SE)
	May 2012	Min-max	Min-max
Population	Long-term (number of years)	*n*	*n*
Cessna Pond	4.33	9.85 (1.20)	0.138 (0.015)
	4.89 (10)	5.50–14.19	0.053–0.205
		8	8
Dickens Hole	0	7.71 (0.401)	0.095 (0.005)
		4.10–7.71	0.060–0.118
		12	12
Flock Pond	0.33	9.04 (0.43)	0.101 (0.012)
		7.42–12.55	0.046–0.194
		11	11
Gambo Bayou	0	9.36 (0.625)	0.139 (0.014)
	6.3 (10)	5.55–12.17	0.094–0.250
		11	11
Happy Endings	13.67	8.84 (0.0537)	0.090 (0.009)
		5.22–11.65	0.054–0.164
		13	13
Lake Jackson	18.67	10.01 (0.433)	0.166 (0.016)
		7.22–12.19	0.114–0.284
		10	10
Lake Overstreet	2	11.41 (0.78)	0.150 (0.014)
	2.17 (4)	5.39–14.03	0.077–0.228
		10	10
McBride Slough	21.67	11.09 (0.348)	0.200 (0.013)
	4.77 (10)	8.79–13.32	0.113–0.283
		15	15
Monk's Corner	0.33	8.06 (0.411)	0.103 (0.008)
		4.41–8.06	0.045–0.171
		18	18
Moore Lake	2	7.19 (0.93)	0.120 (0.032)
	3.48 (10)	4.47–11.68	0.05–0.299
		7	7
Newport Spring	0	13.14 (0.493)	0.208 (0.016)
	1.3 (4)	9.29–15.52	0.068–0.265
		12	12
Shepherd Spring	22.33	10.24 (0.512)	0.163 (0.018)
	3.79 (10)	5.96–13.37	0.062–0.320
		16	16
Trout Pond	7.33	9.14 (0.532)	0.148 (0.013)
	6.81 (10)	7.23–11.24	0.098–0.209
		8	8
Tram Road	5.67	7.64 (0.0929)	0.137 (0.021)
	3.6 (10)	5.46–12.46	0.055–0.240
		7	7
Wacissa River	4.33	9.52 (0.36)	0.120 (0.012)
	46.1 (10)	7.98–9.52	0.058–0.206
		12	12

We dissected each sexually mature male to remove the testis. We freeze-dried the testis and body of each male for 24-hours after which we measured the dry-mass of the testis (testis mass) and the dry-mass of the body (body mass). Body mass and testis mass were both measured on a Mettler–Toledo microbalance to the nearest 0.01 and 0.001 mg, respectively.

We tested whether density varied among populations using an analysis of variance (ANOVA) on log-transformed densities (log adult density + 1). We tested whether populations differed in testis mass using an analysis of covariance (ANCOVA) with population as the factor and body mass as the covariate. Both measurements were log-transformed to meet the assumptions of the ANCOVA. We initially included the population by body mass interaction in the model to assess the homogeneity of slopes assumption. This interaction was not statistically significant; hence, it was removed from the model. We used the least-squares estimated average testis mass from each population as a measure of investment into testis mass adjusted for body mass.

We tested whether average testis mass, adjusted for body mass, was correlated with population density at two different levels. First, we tested whether relative testis mass was correlated with population density using the density estimates from May 2012. Second, for 10 of the 15 populations sampled in May 2012, we tested whether relative testis mass (estimated from the 2012 collection) was correlated with an estimate of long-term average population density (adult density averaged over multiple census years). Estimates of long-term average density for six of the populations (Cessna Pond, McBride Slough, Moore Lake, Shepherd Spring, Trout Pond and Wacissa River) were taken from a previous study (Schrader and Travis 2012). These estimates were made using 10 years of census data. Estimates of long-term average density for the other four populations were calculated using 10 (Tram Road, and Gambo Bayou) and 4 years (Lake Overstreet and Newport Spring) of census data.

We complemented our field survey with an experimental test of whether male investment in testes varies plastically with the risk of SC. This experiment involved two treatment groups. In the first treatment group, we eliminated the potential for sperm competition (NSC) by housing a single adult male with a single female in an 8-L aquarium (*n* = 9). In the second treatment group, we created a high risk of SC by housing five adult males with two females in 8-L aquaria (*n* = 10). The latter treatment simultaneously increased population density and the ratio of males to females, both of which are predicted to increase the level of SC (e.g., Evans et al. 2003; Dziminski et al. 2010). The goal of this experiment was to test whether males are able to alter gonadal investment with increased risk of SC, not to investigate the independent roles of density or sex ratio per se (which are confounded here).

The males used in our experiment were lab-born adult descendants of females collected from three North Florida populations (Moore Lake, Wacissa River, and Wakulla Springs). Assignment of individuals to treatments was made blindly with respect to their population of origin. In order to standardize their prior social environment, all males were housed under conditions eliminating SC for 30-days prior to the start of the experiment (i.e., in male-female pairs). The females used in this experiment had been housed in mixed-sex stock tanks. As we were not interested in measuring effects of SC on females, we did not standardize the social environment they experienced prior to the experiment. We fed each NSC tank 20-mg of ground commercial flake food once a day and the SC tank 2.5 times this amount. We did not increase food levels in direct proportion to the number of individuals in the tank because the SC treatment tanks had male-biased sex ratios and males eat much less than females (J. Travis unpubl. data). We sacrificed males 4 weeks after the initiation of the experiment, dissected each male, freeze-dried the body and testis, and measured body mass and testis mass as described above. Studies of male life history indicate a life expectancy for males after maturity of 4–6 weeks (Leips et al. 2000, J. Travis, unpubl. data), so 4 weeks is a substantial amount of an adult male's expected lifespan.

We tested whether males altered gonadal investment with the risk of SC by comparing the average gonadal somatic index (GSI) values (testis mass/body mass × 100) of each treatment using a *t*-test. The four males in each replicate SC tank are not independent observations and treating them as such would introduce pseudoreplication (sensu Hurlbert 1984). To avoid this problem, we averaged the GSI values for the males in each SC replicate and used the average value for each aquarium as the unit of observation in this analysis. This procedure also avoids the ambiguities inherent in calculating a least-squares adjusted mean testis mass for the males in the SC treatment. It is possible that high levels of intermale competition in our SC treatment tanks could result in the establishment of dominance hierarchies and that the dominant male in each SC tank could either suppress the GSI of the subordinate males or subordinate males may compensate for their disfavored role by increasing investment in sperm production. Under either of these scenarios, we would expect the SC tanks to have more variable GSI values than the NSC tanks. We addressed this possibility by calculating the coefficient of variation in GSI (CV_GSI_) for each SC tank and for the NSC tanks. We then used a one-sample Wilcoxon test to test whether the CV_GSI_ of the SC treatments were different from the CV_GSI_ of the NSC treatment.

## Results

**Figure 2:**
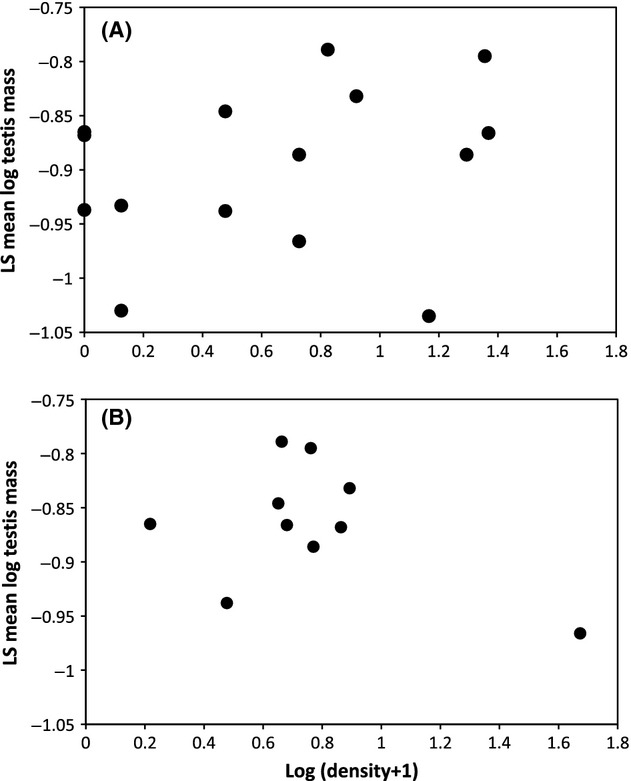
Population density and mean testis mass in *Heterandria formosa* populations. In both panels, the *y*-axis is the least-squares mean log testis mass estimated form the May 2012 collection. In (A) population density (adults per 0.5 m^2^) is the density estimate from May 2012. In (B) population density is the long-term average density from 4 to 10 years of sampling (see Table 1). Density is log-transformed in both panels.

Density varied widely and significantly among populations, from an average of zero adults per 0.5-m^2^ to an average of 23 adults per 0.5-m^2^ (ANOVA; *F*_15,32_ = 3.04, *P* = 0.004). We collected between 7 and 18 mature males per population. The average body mass and testis mass varied by about twofold between the smallest and largest values; the average body mass varied between 7.20-mg in Moore Lake and 13.14-mg in Newport Spring and average testis mass varied between 0.095-mg in Dickens Hole and 0.210-mg in Newport Spring (Table 1). Testis mass increased with body mass (ANCOVA, *F*_15,154_ = 121.60, *P* < 0.0001) and relative testis mass, adjusted for body mass, varied significantly among populations (ANCOVA, *F*_14,154_ = 4.73, *P* < 0.0001). Despite this significant variation among populations in density and relative testis mass, there was no significant correlation between these two variables (Pearson correlation between log relative testis mass and log [density + 1]; *r* = 0.25, *P* = 0.365, *n* = 15; Fig. 2A). Similarly, there was no significant correlation between average population density and relative testis mass among the 10 populations for which long-term density data were available (Pearson correlation between log relative testis mass and log [density + 1]; *r* = −0.41, *P* = 0.24, *n* = 10; Fig. 2B).

**Figure 3:**
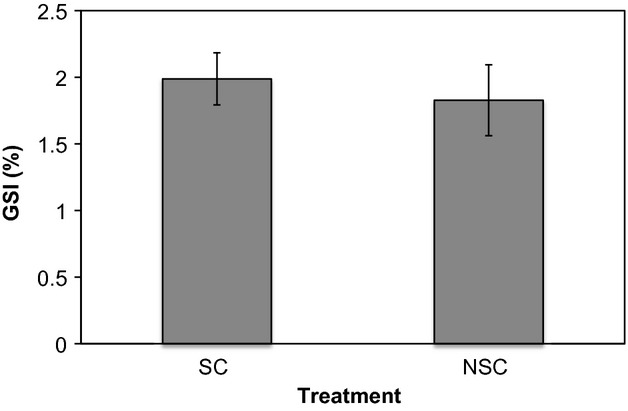
Mean (±1 SEM) gonadosomatic index (GSI) values for males exposed to sperm competition (SC, *n* = 10) and no sperm competition (NSC, *n* = 9).

In the laboratory experiment, there was extensive variation in GSI values with testis mass being between 0.9% and 3.4% of body mass. There was no evidence that GSI values were higher after 1 month of induced SC; the average GSI values for the NSC and SC treatments were both just under 2.0% and were not significantly different from each other (*t*_17_ = 0.49, *P* = 0.63; Fig. 3). There was also no evidence that interactions among males in the SC treatment were skewing the distribution of GSI and biasing our comparison with the NSC treatment. The CV_GSI_ among the NSC replicates was 46.11% and the average CV_GSI_ for the SC treatments was 43.19 (*n* = 10). A one-sample Wilcoxon test revealed no significant difference between these values (*V* = 21, *P =* 0.56).

## Discussion

High population densities should increase the risk of SC in many animals, and several studies have reported positive associations between population density and male gonadal investment, consistent with predictions from SC theory (Gage 1995; Brown and Brown 2003; Long and Montgomerie 2006; Dziminski et al. 2010). In contrast to this pattern, we found no evidence for an association between population density and male gonadal investment despite significant variation among populations in both variables. This was true whether we used contemporary densities or the average historical densities. One might argue that the latter is the better measure of a potential evolutionary pattern, inasmuch as a set of densities in any single year may not reflect the long-term patterns. In this light, it is striking that when we used the long-term densities, the estimated correlation was actually negative, bolstering our confidence that there is no real positive association to be found with more data.

A positive relationship between population density and relative testis mass should be found if two assumptions are met: the risk of SC must increase with population density and high levels of SC must select for increased gonadal investment in males. The absence of an association between density and relative testis mass in our study suggests that one or both of these assumptions are not met in *H. formosa*.

The level of SC increases with population density in several species (Gage 1995; Brown and Brown 2003; Long and Montgomerie 2006; Dziminski et al. 2010), indicating that the first assumption is tenable. However, it remains unclear whether it is true in *H. formosa*. Schrader et al. (2011) found that the proportion of females carrying multiply sired broods (the rate of concurrent multiple paternity [CMP]) was uniformly high across populations that vary considerably in their average densities. One might argue that these data indicate levels of SC are independent of population density. However, rates of CMP are not completely informative about the level of SC for two reasons. First, the rate of CMP measures the frequency with which two males share paternity, not the average number of males whose sperm compete over fertilization within the average female. Second, when brood sizes are small, it is unlikely that every male who mates with a female can be represented in at least one developing offspring. This is a particularly acute challenge in *H. formosa* because small brood sizes are typical in this species (e.g., Schrader and Travis 2009; Schrader et al. 2011). Given the mating system of *H. formosa*, which involves coercive attempts by males and the capacity of females to store viable sperm for much of their adult lifespan, we might expect uniformly high rates of CMP, but very different levels of sire number per female across the different densities. It may be that using genetic methods to enumerate the number of unique males represented in the sperm found in the lumen of a female's ovary will prove the most reliable method for estimating the true level of SC. Such an approach has been used to estimate levels of polyandry, sperm storage, and SC in insects (e.g., Demont et al. 2011).

Whether the second assumption is met in *H. formosa* depends on the nature of SC. Selection will promote increased male gonadal investment if sperm compete numerically (Parker 1982). If sperm do not compete numerically, then selection may favor other sperm traits (e.g., swimming velocity or viability), or even male behaviors that increase competitive fertilization success (e.g., Gasparini et al. 2010; Boschetto et al. 2011). While there are no direct tests of numerical SC in *H. formosa,* there are data suggesting that other factors play important roles in determining which competing males become sires of any single female's offspring, including evidence for sperm precedence (which male mates first: Ala-Honkola et al. 2010) and male persistence (which male attempts to mate more often: Ala-Honkola et al. 2009). Whether high levels of attempted mating influence paternity through increased number of sperm transferred, increased likelihood of sperm transfer, or displacement of rival sperm remains unknown.

We have focused on population density as an ecological variable likely to influence the importance of SC and male gonadal investment in *H. formosa*. However, the populations we studied here differ from one another in many ways and it is possible that some other environmental variable influences the strength of SC or male gonadal investment in these populations. For example, the populations are a mix of springs and ponds that differ in thermal regimes and water chemistry (Leips and Travis 1999; J. Travis, unpubl. data), which may influence testis mass directly (e.g., McManus and Travis 1998). Some of these populations also differ in predation risk (Schrader and Travis 2012), which has been argued to influence the level of SC in guppies, *P. reticulata*. In brief, male guppies in high-predation populations are less likely to court females and more likely to engage in coercive mating attempts. (Luyten and Liley 1985; Magurran and Seghers 1990; Evans et al. 2002; Neff et al. 2008). Higher rates of coercion expose females to mating attempts from more males and thereby increase the rate of SC. Although predation risk varies among *H. formosa* populations (Leips and Travis 1999; Richardson et al. 2006; Schrader and Travis 2012), we do not know if males from populations with historically higher risk of predation thrust more often than do males from populations with historically lower risks of predation. A final possibility is that variation among populations in testis mass may be driven by differences among populations in the average age of males (i.e., gonadal investment may appear to be lower in some populations just because males are younger on average). *Heterandria formosa* males continue to grow after maturity (R. Hale and J. Travis, unpubl. data), however, growth rates vary with environmental conditions, such as water chemistry (R. Hale and J. Travis, unpubl. data), so it is not possible to use male size as a surrogate for male age when comparing ecologically divergent populations. Testing whether variation among populations in testis mass may be driven by differences in male age will require more direct estimates of age-structure.

Our lab experiment increased the risk of SC by simultaneously increasing population density and creating a male-biased sex ratio. However, males did not respond by increasing testis mass. Our results are similar to those of a previous study of *P. reticulata* that examined the separate effects of rearing density and sex ratio on several male traits (Evans and Magurran 1999b). In this experiment, rearing density had no effect on sperm number and GSI remained unaffected by rearing sex ratio. Our experiment was different from Evans and Magurran's (1999b) in that we exposed adult males to different levels of SC while Evans and Magurran manipulated the juvenile environment. Nevertheless, the similarity between our results and Evans and Magurran's study of *P. reticulata* suggest that male poeciliids may not be capable of readily altering gonadal investment in response to short-term changes in the perceived risk of SC. This is surprising because male poeciliids have been shown to alter mating behaviors, ejaculate expenditure, and even gamete characteristics in response to variation in the risk of SC (Evans and Magurran 1999b; Evans and Pilastro 2011; Smith and Ryan 2011). It is possible that males already invest maximally in testes and that they are only able to respond to changes in the risk of SC by altering behavior or ejaculate characteristics (e.g., Evans and Magurran 1999b).

In conclusion, we have shown that relative testis mass varies widely among *H. formosa* populations, but is not obviously related to ecological variables likely to affect the level of SC. We have also shown that *H. formosa* males do not alter their gonadal investment in response to a change in the level of SC they experience. These results suggest that the level of SC does not increase with population density in *H. formosa*, or that the outcome of SC in this species is determined by traits other than testis mass.
